# β-1,3-glucan-lacking *Aspergillus fumigatus* mediates an efficient antifungal immune response by activating complement and dendritic cells

**DOI:** 10.1080/21505594.2018.1528843

**Published:** 2018-10-29

**Authors:** Marion Steger, Marta Bermejo-Jambrina, Teodor Yordanov, Johannes Wagener, Axel A Brakhage, Verena Pittl, Lukas A Huber, Hubertus Haas, Cornelia Lass-Flörl, Wilfried Posch, Doris Wilflingseder

**Affiliations:** a Division of Hygiene and Medical Microbiology, Medical University of Innsbruck, Innsbruck, Austria; b Division of Cell Biology, Biocenter, Innsbruck Medical University, Innsbruck, Austria; c Max von Pettenkofer-Institut für Hygiene und Medizinische Mikrobiologie, Ludwig-Maximilians-Universität München, Munich, Germany; d Department of Molecular and Applied Microbiology, Leibniz Institute for Natural Product Research and Infection Biology (HKI), Germany; e Division of Molecular Biology, Medical University Innsbruck, Innsbruck, Austria; f Department of Microbiology and Molecular Biology, Friedrich Schiller University (FSU), Jena, Germany

**Keywords:** Fungal infection, dendritic cell, complement, T helper cells, β-1, 3-glucan

## Abstract

Complement system and dendritic cells (DCs) form – beside neutrophils and macrophages – the first line of defense to combat fungal infections. Therefore, we here studied interactions of these first immune elements with *Aspergillus fumigatus* lacking ß-1,3-glucans (*fks1_tetOn_^rep^* under repressed conditions) to mechanistically explain the mode of action of echinocandins in more detail. Echinocandins are cell wall active agents blocking β-glucan synthase, making the *A. fumigatus fks1_tetOn_* mutant a good model to study immune-modulatory actions of these drugs. We now demonstrate herein, that complement was activated to significantly higher levels by the *fks1-*deficient strain compared to its respective wild type. This enhanced covalent linking of complement fragments to the *A. fumigatus fks1_tetOn_^rep^* mutant further resulted in enhanced DC binding and internalization of the fungus. Additionally, we found that *fks1_tetOn_^rep^* induced a Th1-/Th17-polarizing cytokine profile program in DCs. The effect was essentially dependent on massive galactomannan shedding, since blocking of DC-SIGN significantly reduced the *fks1_tetOn_^rep^-*mediated induction of an inflammatory cytokine profile.

Our data demonstrate that lack of ß-1,3-glucan, also found under echinocandin therapy, results in improved recognition of *Aspergillus fumigatus* by complement and DCs and therefore not only directly affects the fungus by its fungistatic actions, but also is likely to exert indirect antifungal mechanisms by strengthening innate host immune mechanisms.

**Abbreviations**: C: complement; CR:complement receptor; DC: dendritic cell; iDC: immature dendritic cell; DC-SIGN: Dendritic Cell-Specific Intercellular adhesion molecule-3-Grabbing Non-integrin; ERK: extracellular signal–regulated kinases; JNK : c-Jun N-terminal kinases; MAPK: mitogen-activated protein kinase; NHS: normal human serum; PRR: pattern recognition receptor; Th :T helper; TLR :Toll-like receptor; WT: wild type.

## Background

*Aspergillus (A.) fumigatus* is one of the most prevalent fungal pathogens in immunocompromised patients [1]. Together with macrophages and neutrophils the complement (C) system and dendritic cells (DCs) form a first line antifungal defense by producing inflammatory cytokines, ROS or anaphylatoxins.

*A. fumigatus* was demonstrated to activate the C-system via alternative or classical pathways, depending on morphology and germination status of the fungus [2,3]. Resting conidia represent a poorly immunogenic surface due to hydrophobins (rodlet layer), covering the fungal surface, and DHN-melanin, lying underneath, thereby preventing innate immune recognition [3,4]. Nevertheless resting conidia slowly activate the complement system via the alternative pathway, while swollen conidia or hyphae are mainly recognized by classical C-components [2,3,5]. Less pathogenic *Aspergillus* species, e.g. *A. nidulans*, exhibited higher deposition of C3 molecules on their surface compared to the highly pathogenic species *A. flavus* and *A. fumigatus* [5,6]. Another study revealed lower C3-deposition on wild type *A. fumigatus* conidia when compared to conidia lacking melanin [3,7], thereby pointing to a role of melanin with respect to fungal evasion strategies. The lack in the early melanin enzymes Arp1 and Alb1 was demonstrated to significantly increase C-deposition on *A. fumigatus*, thereby modulating its virulence [3,8]. C3 fragments coating the surface of *A. fumigatus* influence binding to and internalization in phagocytes such as DCs, macrophages or neutrophils. Upon germination fungal cells expose polysaccharides, such as α-1,3–, β-1,3-glucans, galactomannan and galactosaminogalactan [4,9], which ligate other PRRs than CRs on DCs, i.e. TLR2, C-type lectin receptors Dectin-1 and −2 or DC-SIGN [10].

Echinocandines targeting cell wall β-1,3-glucans by inhibiting the β-glucan synthase, cause changes in *A. fumigatus* cell wall composition [11,12]; when treating wild type *Candida glabrata* isolates with caspofungin or micafungin a significantly increased macrophage activation was detected [13]. Thus, changes in cell wall composition of the fungus might also significantly contribute to differential innate and adaptive immune shaping not only via macrophages but also via DCs during echinocandin treatment.

DCs are key regulators of immunity and the most important cells in initiating adaptive fungal immunity [14,15]. During the acute phase of infection cytokines produced by stimulated and matured DCs polarize protective and non-protective T helper (Th) cell responses, T cell subsets providing significant protection against *A. fumigatus* comprise Th1 and Th17 cells, while Th2 cells are associated with a poor outcome of disease [16]. Therefore, immediate secretion of Th1- or Th17-polarizing cytokines by stimulated DCs provides spontaneous triggering of protective antifungal immune responses.

This study aimed at a better understanding of how cell wall components of *A. fumigatus* contribute to the fungal recognition by i) complement and ii) DCs and of whether complement opsonization supports a more efficient sensing by DCs upon invasive fungal infections. Therefore, we investigated *A. fumigatus* wild-type strains as well as mutants devoid of DHN-melanin (*∆pksP*) or ß-1,3-glucan (*fks1_tetOn_* under repressed conditions, *fks1_tetOn_^rep^*) in their cell walls regarding complement (C) activation as well as innate sensing and cytokine up-regulation in iDCs exposed to such non- or C-opsonized fungal conidia.

## Results

### Lack of melanin and β-1,3-glucan favor C3 deposition on A. fumigatus conidia

**Figure 1.**
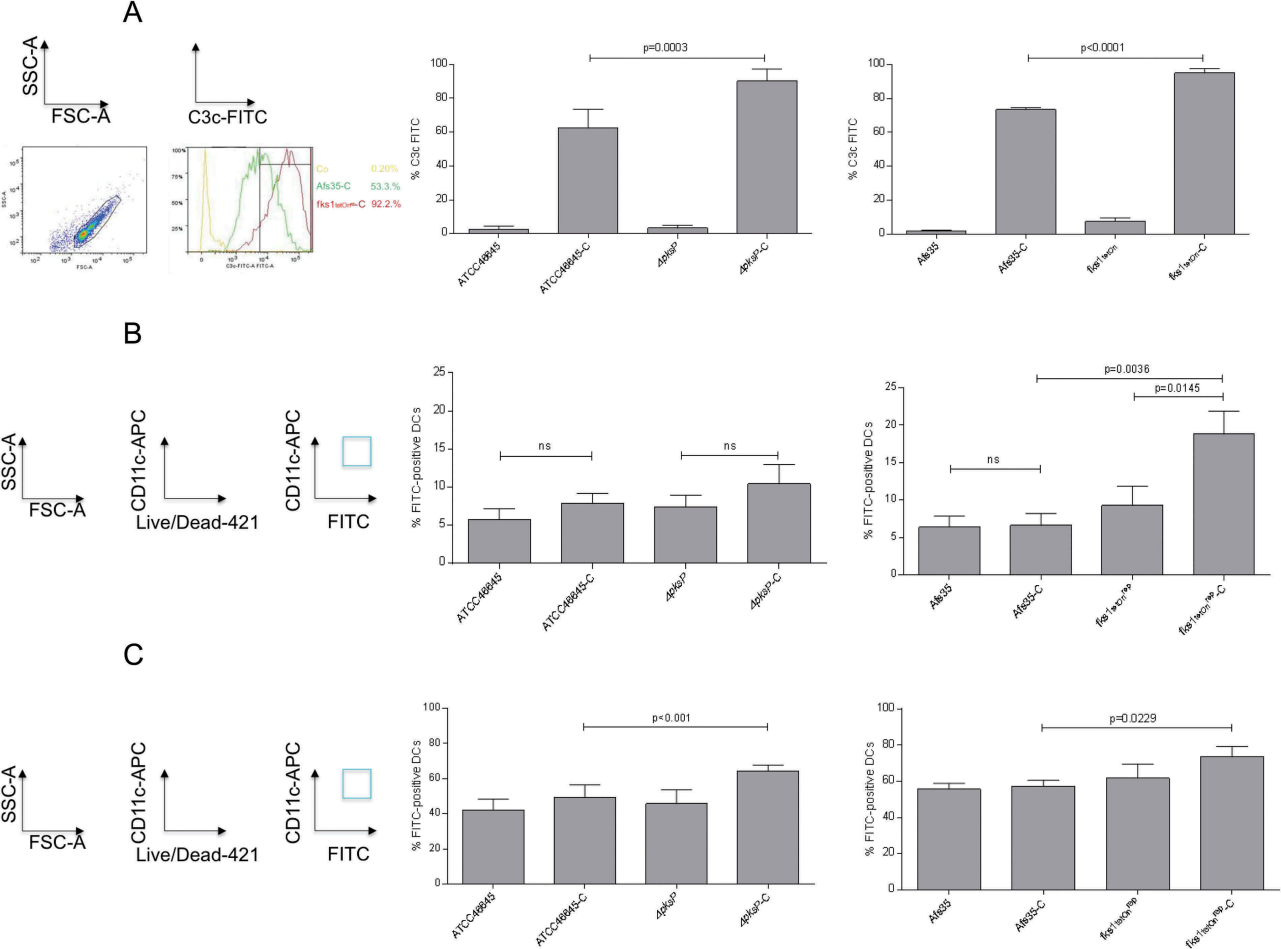
Complement activation and DC interactions depend on cell wall composition. (a) *Melanin- and β-1,3-glucan-lacking strains demonstrate significantly higher surface C3-deposition*. Significantly higher covalently linked C3 levels were detected on *∆pksP* (left) and *fks_tetOn_*^rep^ (right) compared to their respective WT (46645, AfS35). Non-opsonized counterparts served as controls for background C3c-binding. A summary from 6 different experiments (n = 6) is shown for pksP opsonization, and from 10 experiments (n = 10) for fks opsonization. *Binding* (b) *and internalization* (c) *of non- and C-opsonized swollen conidia by DCs*. For binding (4°C) and internalization (37°C) analyzes, percentages of FITC-labelled conidia bound and/or internalized to live CD11c^high^ DCs from at least 5 independent experiments were assessed by multi-color flow cytometry. The gating strategy is depicted on the left. Differences were analyzed by one-way ANOVA using Bonferroni´s post test for multiple comparisons.

Significantly higher C3c deposition (mean 89.8% C3^+^ conidia, Figure 1(a), left) was observed for *∆pksP* compared to WT ATCC46645-C (59.2% C3^+^ conidia, Figure 1(a), left), which is in agreement with published data [17]. Also the *fks1_tetOn_^rep^* mutant showed a significantly higher complement deposition compared to its respective wild type AfS35 (94.9% vs. 76.7% C3^+^ conidia, Figure 1(a), right), while in all non-opsonized controls only background C3 deposition was analyzed (Figure 1(a)). C3 deposition on the *fks1_tetOn_^rep^* was even significantly higher compared to *∆pksP* (p = 0.0384). We here found that lack of either melanin or β-1,3-glucan results in significantly higher C3 coating of the fungal conidia.

### Complement-opsonized conidia exert improved recognition by DCs

To further characterize binding of non- and opsonized WT and mutant conidia, co-cultures of DCs and FITC-labeled conidia (MOI = 2) were incubated for 6h at 4°C [18]. At 4°C, DCs just bind but do not internalize fungal pathogens – again we used FACS analyses and gated on live CD11c^high^ DCs. CD11c^high^/FITC^high^ double-positive cells were used to determine percentages of conidia attached to the cells (Figure 1(b), left). We found highest levels of *fks1_tetOn_^rep^-C* (mean 20% FITC-positive DCs, Figure 1(b), right) attached to DCs followed by *∆pksP-C* (mean 10% FITC-positive DCs) (Figure 1(b), left) and *fks1_tetOn_^rep^* (mean 9% FITC-positive DCs, Figure 1(b), right). Therefore, C-opsonization of conidia enhanced DC binding in case of both mutant strains, but not the WT conidia (Figure 1(b)).

Regarding internalization, which was performed for 6h at 37°C, significantly higher combined binding and internalization of *∆pksP*-C were detectable compared to its opsonized WT, which is due to the higher activity of the cells at this temperature (Figure 1(c), left, 49.4% ATCC46645-C vs. 64.3% *∆pksP*-C). Highest binding/internalization was measured using *fks1_tetOn_^rep^-C* (Figure 1(c), right, 73.5%) but also non-opsonized *fks1_tetOn_^rep^* demonstrated a slightly higher, but non-significant up-take into DCs compared to its WT (Figure 1(c), right, 59.8%). In concordance with the highest C3-deposition on *fks1_tetOn_^rep^* devoid of β-1,3-glucan, we here demonstrated the highest binding and internalization by DCs.

### Efficient internalization of ∆pksP-C and fks1_teton_-C is mediated by CR3 and CR4

**Figure 2.**
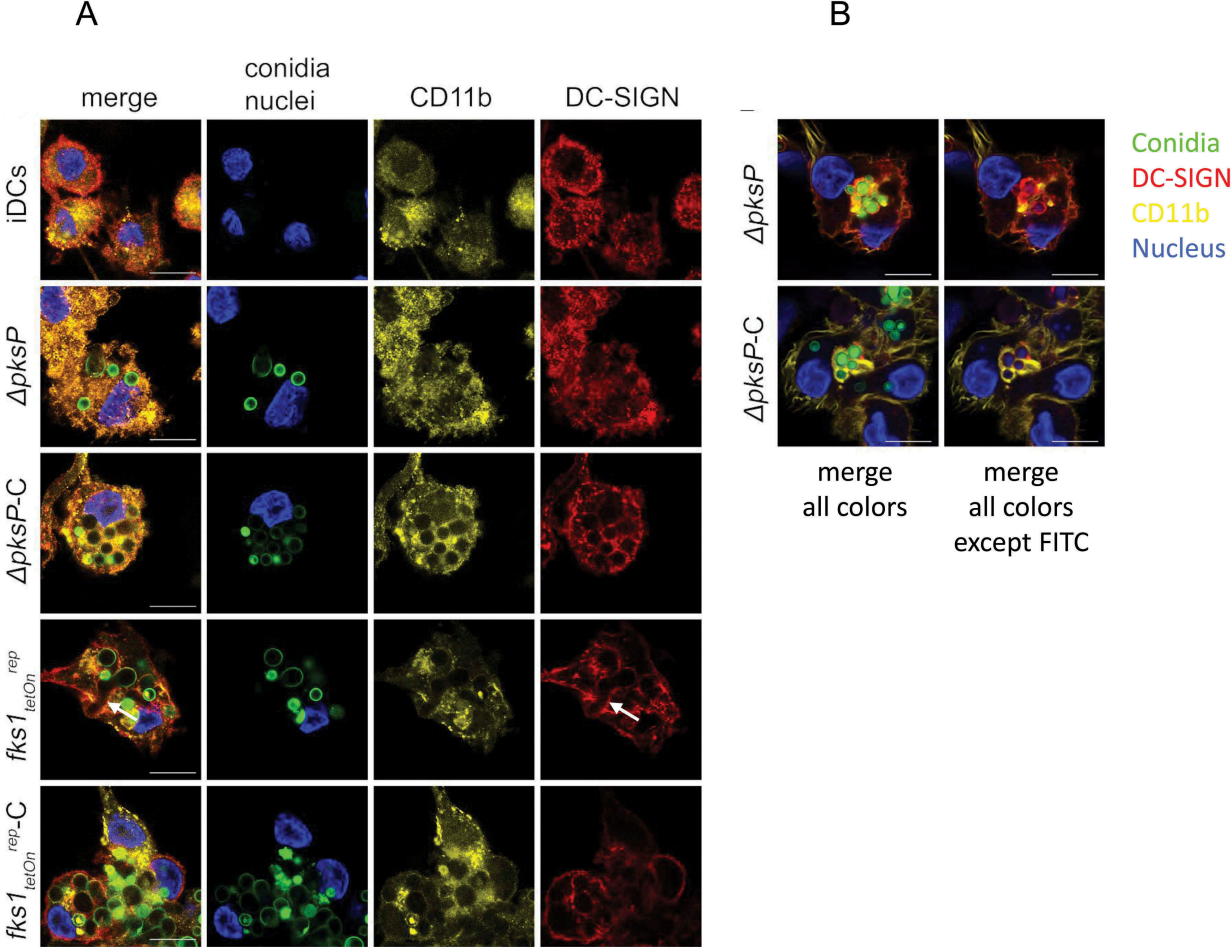
C-opsonized conidia show an improved up-take into DCs. After 6h of co-culture at 37°C, CD11b (yellow) in DCs co-localized with opsonized fungal conidia (green) (a and b, panels *∆pksP-C* and *fks_tetOn_^rep^-C)*, whereas DC-SIGN (red) was found to be highly involved in binding of germlings (a, arrow) and non-opsonized conidia (b, upper panel). Higher amounts of opsonized fungal conidia were bound/internalized by DCs compared to non-opsonized conidia. A more detailed analysis of the specific involvement of DC-SIGN (red) in internalizing non-opsonized and CD11b (yellow) in taking-up opsonized conidia is depicted in Figure 2(b). Left panels depict all fluorescence channels (green: conidia, red: DC-SIGN, yellow: CD11b, blue: nucleus), while in right panels the FITC channel was switched off to emphasize the involvement of DC-SIGN in internalization of non-opsonized and CD11b in up-take of opsonized fungal conidia. Data shown are representative of 3 independent experiments. Scale bar represents 10µm.

To further outline the importance of CD11b, the α-chain of CR3, and CD11c, the α-chain of CR4, in enhanced opsonized conidia uptake, we next performed confocal microscopic analyses under the same conditions as described for the internalization assay (6h, 37°C). After 6h of iDC/fungi co-culture, CD11b clearly co-localized with C-opsonized germlings (*ΔpksP*-C and *fks1_tetOn_^rep^*-C, Figure 2(a)). In contrast, iDCs and *ΔpksP*-DCs demonstrated an even distribution of CD11b (Figure 2(a)). DC-SIGN co-localized with CR3 but was mainly involved in up-take of germinating conidia as found for *fks1_tetOn_^rep^* (arrows, Figure 2(a)). Figure2(b) shows the receptor-dependent phagocytosis of non- and C-opsonized conidia into DCs in more detail – while non-opsonized *ΔpksP* were surrounded by DC-SIGN (red) after 6h incubation period, *ΔpksP*-C demonstrated a clear CD11b covering within DCs (yellow). Comparable to CD11b distribution, CD11c also co-localized to higher levels with opsonized fungal strains (*ΔpksP*-C and *fks1_tetOn_^rep^*-C, Suppl. Figure 1), while illustrating an even distribution after iDC exposure to the non-opsonized counterparts (*ΔpksP* and *fks1_tetOn_^rep^*, Suppl. Figure 1). DC-SIGN again recognized germinating conidia (*fks1_tetOn_^rep^*, arrows, Suppl. Figure 1), but also co-localized to low levels with CD11c after addition of C-opsonized mutants (*ΔpksP*-C and *fks1_tetOn_^rep^*-C, Suppl. Figure 1). In all these experiments, and also identified by flow cytometry, higher amounts of conidia were internalized by stimulated DCs when they were opsonized. Altogether we conclude that both CR3 and CR4 play a role in efficient binding and internalization of C-coated spores.

### Both CR3 (CD11b/CD18) and CR4 (CD11c/CD18) contribute to phagocytosis of complement-opsonized fungi

**Figure 3.**
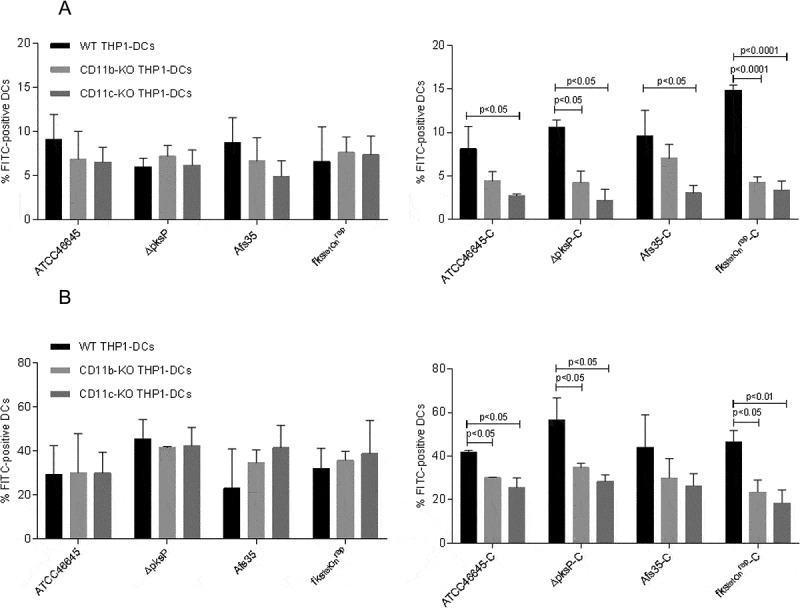
Binding and Internalization of complement-opsonized conidia are impaired upon knock-out of either CD11b or CD11c. CD11b KO- (gray) and CD11c KO THP1-DCs (dark gray) or their respective WT THP1-DCs (black) were incubated for 6h at 4°C (a) or 37°C (b) with non-opsonized (*left*) or opsonized (*right*) fungal strains and binding (a) or internalization (b) were analyzed by flow cytometry. While all non-opsonized fungal preparations showed similar binding (a) and internalization (b) to THP1 KO and WT DCs (*left*), knock-out of CD11b (gray) and CD11c (dark gray) resulted in an impaired binding (a) and internalization (b) process of opsonized fungi (*right*). Highest impairment in binding (a) and internalization (b) were analyzed for the opsonized mutants *∆pksP-C* and *fks_tetOn_^rep^-C* using both, CD11b KO (*right*, gray) and CD11c KO THP1-DCs (*right*, dark gray) compared to WT THP1-DCs (*right*, black). For both analyzes, percentages of FITC-labeled conidia bound and/or internalized by live CD11c^high^ DCs from 3 independent experiments were assessed by multi-color FACS and statistical significance evaluated by one way ANOVA using Bonferroni´s post test for multiple comparisons.

After knocking out CD11b (α-chain of CR3) and CD11c (α-chain of CR4) on THP1-DCs, binding (Figure 3(a)) and internalization (Figure 3(b)) of C-opsonized WT strains (ATCC46645-C, AfS35-C) and mutant strains (*∆pksP-C* and *fks1_tetOn_^rep^-C)* were significantly reduced (Figure 3(a,b)). Binding and internalization of non-opsonized strains were not affected independently on the fungal surface (Figure 3(a,b)). These analyses highlight that up-take of opsonized fungal spores is dependent on CR3 and CR4 and that these receptors act in a coordinated fashion with the ability to substitute each other.

### fks1_tetOn_^rep^ induces enhanced production of pro-inflammatory cytokines from DCs

**Figure 4.**
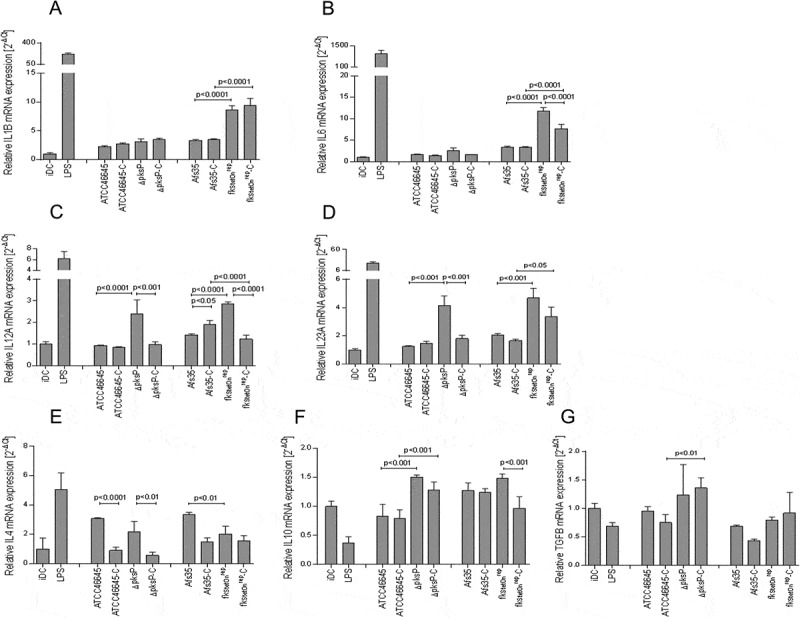
A to G Increased expression of pro-inflammatory cytokines in DCs exposed to opsonized swollen *∆pksP* and *fks1_tetOn_^rep^* conidia. *fks_tetOn_^rep^* skewed DCs into polarizing inflammatory Th1 and Th17 by induction of increased IL1B (a), IL6 (b), IL12A (c) and IL23A (d) mRNA (expression and the Th1-priming capacity of DCs was abrogated in presence of complement fragments on the surface of this mutant (c). Further, we found that lack of melanin also elevated IL12A (c) and IL23A (d) mRNA expression, but showed only marginal induction of IL1B (a) or IL6 (b) mRNA expression. WT conidia induced slight but non-significant elevations of the pro-inflammatory cytokines compared to non-treated iDCs. LPS-treated DCs served as positive controls for stimulating pro-inflammatory cytokines in DCs (a to d). IL10 (f) and TGFB (g) mRNA levels were only slightly changed compared to iDCs, while IL4 (e) mRNA levels were up-regulated to some extent in DCs exposed to the WT strains, but down-modulated by *∆pksP* and *fks_tetOn_^rep^* or complement-opsonization (e). The summary of 3 independent experiments are demonstrated. Significances were evaluated by one way ANOVA using Bonferroni´s post test for multiple comparisons.

Since cytokines play an essential role in initiating and shaping T helper cell responses, we next performed multiplex PCR analyses (Figure 4(a-g)) to detect expression of IL1B (A), IL6 (B), IL12A (C), IL23A (D), IL4 (E), IL10 (F) and TGFB (G) in DCs exposed to the non-/C-opsonized fungal WT and mutant strains. *fks1_tetOn_^rep^* significantly up-regulated IL1B (A), IL6 (B), IL12A (C), and IL23A (D) mRNA compared to the WT AfS35 and iDC control cells, thereby inducing a Th1/Th17-polarizing cytokine pattern in DCs (Figure 4). IL1B (A), IL6 (B), IL12A (C), and IL23A (D) mRNA levels were elevated up to 15-fold in *fks1_tetOn_^rep^*-DCs (Figure 4). Similar IL1B (A) mRNA levels were detected in *fks1_tetOn_^rep^*- and *fks1_tetOn_^rep^-C*-loaded DCs (Figure 4). *fks1_tetOn_^rep^-C*-exposed DCs down-modulated IL6 (B) and IL23A (D) mRNA levels compared to *fks1_tetOn_^rep^*-DCs, but levels induced were still significantly higher compared to Afs35-C-DCs (Figure 4). IL12A mRNA expression levels were significantly decreased in *fks1_tetOn_^rep^-C*-exposed DCs compared to Afs35-C- and *fks1_tetOn_^rep^*-DCs (Figure 4(c)). Thus, C-opsonized *fks1_tetOn_^rep^* mediated a Th17-, but no Th1-polarizing cytokine profile in DCs. In contrast to the increased pro-inflammatory cytokine expression, IL-10 and TGF-β expression levels were only marginally altered in both *fks1_tetOn_^rep^* and *fks1_tetOn_^rep^*-C-DCs compared to DCs treated with the respective WT (Afs35, Afs35-C) or in immature DCs (iDCs) (Figure 4(f-g)). IL-4 mRNA expression was significantly lowered by the *fks1_tetOn_^rep^* mutant in DCs compared to its wild type and levels were similar to iDCs (Figure 4(e)). C-opsonization of fungi prevented up-regulation of IL-4 independent of the strain used (Figure 4(e), 3^rd^ panel, left, ATCC46645-C, *ΔpksP*-C, Afs35-C, *fks1_tetOn_^rep^*-C).

IL12A (C) and IL-23A (D) mRNA expression levels were also significantly up-regulated in DCs exposed to *ΔpksP* in comparison to its respective WT. As observed using C-opsonized *fks1_tetOn_^rep^, ΔpksP-C* significantly dampened the inflammatory response in DCs compared to their non-opsonized counterpart. IL-1B and IL-6 mRNA expression levels were slightly induced in DCs by the *ΔpksP* mutant compared to its WT and independent of the opsonization pattern. TGFB mRNA was slightly induced in DCs treated with the *ΔpksP* mutant.

These results imply a role for β-1,3-glucan to hinder the DC-mediated polarization of inflammatory Th1 and Th17 and to favor the induction of Th2- or Treg- cells. Further, we found that lack of melanin also results in elevated IL-12A and IL-23A mRNA expression, while IL-1B or IL-6 mRNA expression was not altered. These experiments emphasize the considerable impacts of cell wall components with respect to downregulation or ablation of functional antifungal T helper cell responses.

### fks1_tetOn_^rep^*-C activates MAPK and NFκB in DCs*

**Figure 5.**
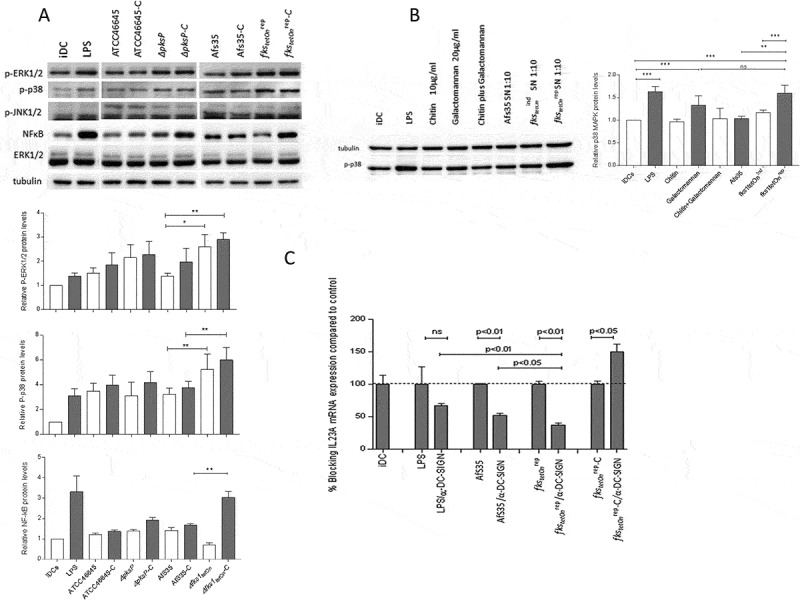
Differential induction of MAPKs and NFκB in DCs upon stimulation with *A.fumigatus* WT and mutant strains. (a) Phosphorylation patterns of ERK1/2 (42 kDa, 44 kDa), p38 MAPK (40 kDa) and NFκB in DC lysates after 1h exposure to non- and C-opsonized fungal WT and mutant conidia (ratio 1:5) were analyzed by Western Blot. Non-stimulated iDCs were used as negative, LPS-exposed DCs as positive controls for DC stimulation. We found that DCs exposed to *fks1_tetOn_^rep^* and *fks1_tetOn_^rep^*-C mediated in particular increased p38 MAPK phosphorylation compared to WTs. ERK1/2 were also activated to higher levels compared to their WT counterparts. Using *ΔpksP* and *ΔpksP-*C ERK1/2 was activated comparable to the LPS control. p38 MAPK phosphorylation was marginal. NFκB was activated in particular by C-coating of the *fks1_tetOn_^rep^*. Tubulin and ERK1/2 were used as controls for protein loading. One representative Western Blot out of three and histogram plots from all donors are depicted. (b) p38 MAPK phosphorylation was assessed in lysates from DCs treated with either 10µg/ml chitin (3^rd^ lane) or 20µg/ml galactomannan (4^th^ lane) alone, in combination (5^th^ lane) or SNs from AfS35 WT (6^th^ lane), *fks1_tetOn_^ind^* (7^th^ lane) or *fks1_tetOn_^rep^* (8^th^ lane). LPS-stimulated (2^nd^ lane) and iDCs (1^st^ lane) served as positive or negative controls, respectively. One representative Western Blot out of three independent experiments is demonstrated, a summary of all is shown in the histogram plot. (c) Blocking DC-SIGN most significantly reduced IL23A mRNA expression upon exposure to *fks1_tetOn_^rep^* compared to WT- or LPS-exposed DCs. In contrast, DCs loaded with complement-coated swollen *fks1_tetOn_^rep^* conidia did not show any decrease in IL23A mRNA expression upon DC-SIGN blocking. The experiment was repeated three times and one representative donor is depicted.

Next we studied phosphorylation patterns of MAPK in DCs after exposure to non- and C-opsonized fungal WT and mutant strains. Non-stimulated iDCs were used as negative controls for expression and activation, LPS-stimulated DCs as positive controls and ERK1/2 and tubulin antibodies as controls for protein loading (Figure 5(a)). In concordance with the results obtained by relative quantification of cytokine expression, we found that DCs exposed to *fks1_tetOn_^rep^* and *fks1_tetOn_^rep^*-C induced p38 MAPK and ERK1/2 phosphorylation to significantly higher levels compared to their counterparts (AfS35, AfS35-C; Figure 5(a)). Using *ΔpksP* and *ΔpksP-*C-loaded DCs only modest p38 MAPK activation was detectable compared to controls, while ERK1/2 were elevated by these mutants (Figure 5(a)). Results obtained by these immunoblot experiments are in accordance with the data obtained by real-time PCR assays. To further investigate the correlation of opsonization of fungal mutants *ΔpksP* and *fks1_tetOn_^rep^* with higher Th17-polarizing capacity, we also analyzed their influence on NFκB activation. We found that beside LPS-stimulated DCs, particularly *fks1_tetOn_^rep^*-C-exposed DCs showed the highest level of NFkB activation (Figure 5(a)). These experiments clearly demonstrate the induction of various inflammatory cytokines in DCs exposed to *A. fumigatus* devoid of β-1,3-glucan or melanin via differential MAPK and NFκB activation profiles.

### Galactomannan promotes p38 MAPK activation and induction of a pro-inflammatory cytokine profile in DCs

To examine the mechanisms underlying the significantly increased Th17-polarizing capacity of *fks1_tetOn_^rep^* on DCs, we further evaluated the factors responsible for spontaneous protective antifungal immune responses. We next determined p38 MAPK phosphorylation, which is activated within DCs to trigger their Th17-polarizing potential. DCs were treated with either chitin or galactomannan alone, in combination and SNs from the AfS35 WT, *fks1_tetOn_^ind^* (under induced conditions) and *fks1_tetOn_^rep^* (Figure 5(b)). Significantly higher p38 MAPK phosphorylation levels were induced using LPS, galactomannan (20µg/ml) and *fks1_tetOn_^rep^* SN (Figure 5(b)).

Contrary to that, AfS35 SN, *fks1_tetOn_^ind^* SN, chitin (10µg/ml) or a combination of chitin/galactomannan only slightly changed p38 MAPK phosphorylation compared to iDC controls (Figure 5(b)). These experiments indicate that galactomannan contributes to the pro-inflammatory signature mediated by DCs, but not chitin or a combination of both compounds do not. To further investigate this, we applied galactomannan (20µg/ml), AfS35 and *fks1_tetOn_^rep^* SNs to DCs and compared their pro-inflammatory profile to chitin-treated DCs by relatively quantifying IL-1B, IL-6, IL-23A and IL-10 mRNA (Suppl. Figure 2). We found that in particular the *fks1_tetOn_^rep^* SN induced up-regulation of IL-1B (upper, left), IL-6 (lower, left) and IL-23A (upper, right) mRNA levels, while IL-10 (lower, right) mRNA levels were not affected compared to control cells.

### *Blocking DC-SIGN significantly decreases* fks1_tetOn_^rep^*-mediated up-regulation of pro-inflammatory mRNA expression in DCs*

Previously, galactomannan was demonstrated to mainly signal via DC-SIGN [19,20] while β-1,3-glucan is recognized by Dectin-1 [21,22]. Since we suppose that the inflammatory effects induced in DCs by *fks1_tetOn_^rep^* are based on galactomannan-mediated mechanisms, we next hindered interactions of the various strains using a blocking anti-DC-SIGN antibody. After inhibiting DC-SIGN-mediated interactions, we found that IL-23A mRNA expression was most significantly reduced in DCs exposed to *fks1_tetOn_^rep^* (63% inhibition, Figure 5(c)) compared to its AfS35 WT strain (45% inhibition, Figure 5(c)) or LPS-exposed DCs (40% inhibition, Figure 5(c)). Blocking DC-SIGN did not reduce IL-23A mRNA expression when DCs were exposed to C-opsonized *fks1_tetOn_* but significantly enhanced its expression (Figure 5(c)). Blocking experiments using an anti-DC-SIGN antibody revealed galactomannan being the driving force to initiate pro-inflammatory events in DCs.

## Discussion

To evaluate the effects of cell wall compounds with respect to spontaneous induction of innate immune responses, we here compared *A. fumigatus* WT strains and their respective mutants *fks1_tetOn_^rep^* and *ΔpksP* in their capacity to bind C3 and subsequent interactions of differentially opsonized WT and mutant conidia with human DCs.

In particular, we were interested in the conditional *fks1_tetOn_* mutant, which has the endogenous *fks1* promoter replaced by the doxycycline-inducible tetON promoter [11]. In *A. fumigatus* WT strains β-(1,3) glucan surface exposure is highest on swollen conidia and early germ tubes, and declines on hyphae, emphasizing key differences in cell wall composition among morphotypes [9]. We could show that not only lack in melanin but β-1,3-glucan fosters significantly higher complement deposition on fungal conidia, indicating that polysaccharides in the DHN-melanin or β-1,3-glucan mutants are more efficiently recognized by the thioester-containing domain (TED) of complement proteins [23] or that the WT strains exert higher complement-degrading or -inactivating capacities [5,24]. Chitin found to significantly higher levels in the cell wall of the *fks1_tetOn_^rep^* mutant [11] was shown to activate the alternative C pathway [25]. Therefore, we supposed that chitin might be the driving force for the enhanced complement deposition on the *fks1_tetOn_^rep^* mutant.

Increased up-take of C-opsonized swollen conidia was mediated via both, CR3 and CR4, as detected by CRISP-Cas9-deletion mutants for CD11b and CD11c. As described earlier, non-opsonized fungal strains attached to DCs preferentially via C-type lectin receptors, i.e. dectin-1 or DC-SIGN [21]. DC-SIGN ligation was shown to simultaneously stimulate other PRRs, e.g. Toll-like receptor (TLR) 2, TLR4 or dectin-1. The cooperation of these intracellular pathways consequently enhanced TLR-mediated IL-12A, IL-12B and IL-6 transcription [26–28], thereby promoting a protective antifungal Th1 response. Not only Th1 but also Th17 cells were activated via DC-SIGN ligation, since indirectly also IL-1B is induced in DCs upon the DC-SIGN/mannose interaction domain via RAF1 activation [26,28]. Thereby, signaling by mannose-ligated DC-SIGN activates a pro-inflammatory cytokine program, a fact we also found in our study using the β-glucan lacking mutant *fks1_tetOn_*^rep^. This mutant induced significantly higher transcription of IL-1B, IL-6, and IL-12A as well as IL-23A mRNA expression compared to its corresponding WT. Not only the β-1,3-glucan- but also the melanin-lacking mutant mediated transcription of both, IL-12A and IL-23A mRNA due to their higher visibility for the immune system compared to wildtype conidia. In contrast IL-1B and IL-6 mRNAs were transcribed to lower levels by the *∆pksP* mutant, thereby pointing to a role of cell wall composition with respect to the magnitude of inflammatory immune response. Dampening of in particular a Th1 polarizing cytokine pattern was noted upon opsonization of the *fks1_tetOn_*^rep^ mutant. This implicates that the *fks1_tetOn_*^rep^ strain is a very strong inducer of a Th1/Th17-polarizing cytokine pattern; its opsonized counterpart more likely mediates a Th17-polarizing program in DCs. We earlier showed that complement-opsonization of HIV-1 mediates polarization of naïve CD4 + T cells into Th17 cells and IL-1β, IL-6 and IL-23 mRNA – secreted protein levels correlated in this study [29]. Induction of a Th17-polarizing cytokine pattern by *fks1_tetOn_*^rep^–C may arise from cross-talk between CR3 or CR4 with DC-SIGN as shown by confocal microscopic analyses.

In contrast to *fks1_tetOn_*^rep^, complement opsonization of the DHN-melanin-lacking strain *∆pksP* caused dampening of both IL-12 and IL-23A mRNA levels. Dampening of pro-inflammatory immune responses by complement opsonization was described earlier in the literature [14], but is also dependent on the surface composition of the fungal strain as described herein. p38 MAPK phosphorylation was shown to be activated by various cytokines as IL-1β [30] and in our analyses we detected the highest p38 MAPK activation using *fks1_tetOn_^rep^* and *fks1_tetOn_^rep^* – C, which also showed the highest expression levels of IL-1B mRNA. mRNA expression of IL-1B as well as activation of p38 MAPK were significantly lower using *∆pksP*. C-opsonization triggered NFκB activation particularly in DCs exposed to the C-opsonized mutants.

Since DC-mediated inflammatory responses were highest in *A. fumigatus* lacking β-1,3-glucan, we aimed to characterize the mechanism(s) responsible for this stronger protective antifungal immunity.

The composition of the *A. fumigatus* cell wall, which is built up of covalently bound β-glucan, chitin, galactomannan and α-glucan, makes recognition by pattern recognition receptors (PRRs) possible [31]. The C-type lectin receptor Dectin-1 was demonstrated to be crucial for recognition of β-glucan expressed at the budding scars of pathogenic or opportunistic fungi, e.g. *Candida* or *A. fumigatus* [19]. DC-SIGN has been shown to be involved in recognition of fungal PAMPs, expressed by CD1c^+^ DCs and inflammatory DCs and on certain macrophage subsets [32]. Binding of fungal PAMPs, i.e. galactomannan, to DC-SIGN initiates simultaneous stimulation of other PRRs, including TLR2, TLR4 or dectin-1. The cooperation of these intracellular pathways consequently enhances TLR-mediated transcription of IL12A, IL12B and IL6 [26–28], thereby promoting a protective antifungal Th1 response [32]. Not only Th1, but also Th17 cells are activated via DC-SIGN ligation, since indirectly also IL1B is induced in DCs upon the DC-SIGN/mannose interaction domain via RAF1 activation [26,28]. Thereby, signaling by mannose-ligated DC-SIGN activates a pro-inflammatory cytokine program, which is consistent with our studies.

Lack in β-1,3-glucan was compensated by massive galactomannan shedding, and this polysaccharide more likely induces a Th1-/Th17 signature via DC-SIGN cross-linking on inflammatory DCs [19,20]. We found 15-fold higher galactomannan levels in *fks1_tetOn_^rep^* SNs – these SNs also efficiently activated p38 MAPK. Several groups using purified cell wall molecules could show, that different receptors recognize cell wall mannan and for example DC-SIGN was able to discriminate between *C. albicans* and *Saccharomyces cerevisiae* mannans [33]. In our study blocking experiments using an anti-DC-SIGN antibody revealed that DC-SIGN is the main – but not sole – target for *fks1_tetOn_^rep^*-mediated induction of a pro-inflammatory immune response. In contrast, AfS35 or *fks1_tetOn_^rep^*-C mainly signaled via receptors other than DC-SIGN.

CR3 (CD11b/CD18) as well as several scavenger receptors (CD5, CD36 and SCARF1) have also been demonstrated to bind β-1,3-glucan and to play an important role in antifungal immunity [34]. These effects are supposed to be even enhanced in our study using a complement-opsonized strain.

β-1,3-glucan-lacking *A.fumigatus* promoted improved complement and DC activation via various mechanisms. On the one hand, complement was efficiently activated by the mutant and covalently fixed at the surface of fungal conidia, probably via the increased cell wall chitin levels. These in turn resulted in better recognition by DCs via CR3 (CD11b/CD18) and CR4 (CD11c/CD18). On the other hand, we here demonstrated DCs move towards a pro-inflammatory, Th1- and Th17-polarizing profile in a DC-SIGN-dependent manner. Our results using β-1,3-glucan-lacking *A. fumigatus* may explain novel immune-modulatory effects mediated by echinocandins, the newest class of antifungals used in the clinics. These drugs impairing cell wall β-1,3-glucans [11,12] may not only act directly on fungal pathogens, but also exert efficient complement- and DC-modulatory mechanisms due to elevated levels of cell wall chitin and elevated galactomannan release.

## Methods

### Ethics statement

Written informed consent was obtained from all blood donors by the Central Institute for Blood Transfusion & Immunological Department, Innsbruck, Austria. All participants were adults; no minors were enclosed in this study. The use of anonymized leftover specimens for scientific purposes was approved by the Ethics Committee of the Medical University of Innsbruck.

### Generation of human monocyte-derived DCs and THP1-DCs

Monocytes were isolated from blood of healthy donors as described before [35,36]. Blood for the monocyte isolation was received by the Central Institute for Blood Transfusion & Immunological Department, Innsbruck, Austria. Briefly, PBMCs (peripheral blood mononuclear cells) were isolated from blood of healthy donors, obtained by a density gradient centrifugation using a Ficoll Paque Premium (GE Healthcare) gradient. After washing, CD14^+^ monocytes were isolated from PBMCs using anti-human CD14 Magnetic Beads (BD) – the purity of the isolated cells was at least 98%. Monocytes were stimulated by addition of IL-4 (200U/ml) and GM-CSF (300U/ml) for five days to generate immature moDCs (iDCs), which were used for all further experiments. Non-stimulated iDCs were used as controls for all experiments using moDCs. THP1-WT and KO DCs were generated from the respective THP1 cells by addition of IL-4 (200U/ml), GM-CSF (300U/ml) and TNF-α (10ng/ml).

### Genome editing using CRISPR/Cas9-mediated depletion of CD11b and CD11c

For CRISPR/Cas9-mediated depletion, guide RNA (gRNA) targeting sequences for CD11b (5´- GCCGTAGGTTGGATCCAAACAGG-3´) and CD11c (5´-GTAGAGGCCACCCGTTTGGTTGG-3´) were selected using an online prediction tool (CRISPR Design; Zhang lab [37]). gRNAs were cloned into a lentiCRISPRv2 vector via BsmBI restriction sites. lentiCRISPRv2 was a gift from F. Zhang (Massachusetts Institute of Technology, Cambridge, MA; Addgene plasmid 52961; [38]). Lentiviral transduction was performed as described in the following paragraph. Depletion efficiency was verified via FACS and live CD11b-KO or CD11c-KO-THP1 cells were isolated by the Core Facility FACS Sorting at the Medical University of Innsbruck.

### Lentiviral transduction

Lentiviral plasmids were co-transfected with Lipofectamine LTX (Invitrogen, cat 15338100) together with pVSV-G and psPAX2 into the HEK293LTV producer cell line. Supernatants containing viral particles were harvested 48h and 72h post transfection, filtered using a 0.2µm filter and directly used to transduce target THP1 cells with 5µg/ml Polybrene (Sigma-Aldrich, cat TR-1003-G). After seven days transduced cells were selected using puromycin (5µg/ml, Sigma-Aldrich, cat SBR00017) and the depletion efficiency of CD11b-KO- and CD11c-KO THP-1 cells was analyzed by flow cytometry (Suppl. Figure 3). Single-cell clones of the specific KO cells were generated after FACS sorting.

### Fungal strains and growth conditions

Fungal strains comprized the clinical isolate ATCC46645 (WT) [39] and its mutant *ΔpksP*, lacking the polyketide synthase required for DHN-melanin synthesis [39]. Further we used WT AfS35 [40] and its mutant *fks1_tetOn_^rep^* [11]. Fks1 as ß-1,3-glucan synthase is an integral plasma membrane protein and provides the ß-1,3 glucan scaffold for other polysaccharides of the cell wall, e.g. galactomannan [41–43]. In the conditional *fks1_tetOn_^rep^* mutant the endogenous *fks1* promoter was replaced by the doxycycline-inducible tetON promoter [44]. Growth media with doxycycline (0.5µg/ml) allowed WT-like growth characteristics (induced (ind) conditions, *fks1_tetOn_^ind^*), which is shown in Suppl. Video 1. Without doxycycline and under repressed conditions, germinated conidia display growth alterations like delayed growth, hyperbranching or cell lysis due to the lack of *fks1* (Suppl. Video 2).

The strains were cultivated on *Aspergillus* complete medium (ACM) agar plates for 5 d at 37°C, whereas for the *fks1_tetOn_* mutant strain the ACM was supplemented with 0.5µg/ml doxycycline under induced conditions [11]. Conidia were harvested with spore buffer (PBS with 0.1% Tween20), filtered through a 5µm filter (PARTEC, CellTricks) to remove hyphae, counted by a hemacytometer and suspended at a concentration of 1*10^7^ spores per ml. For all analyzes, repressed conditions (*fks1_tetOn_^rep^)* were induced by pre-incubating conidia for 3h in *Aspergillus* minimal medium (AMM) without doxycycline prior to further treatments (opsonization, DC exposure) for another 1h to 6h.

### Opsonization and FITC-labeling of conidia

Opsonization was performed by adding 10% NHS (normal human serum from healthy donors) as complement (C) source to pre-incubated swollen conidia for 30 min and another 10% for 30 min. To quantify C3c deposition by flow cytometry (FACS Verse, BD Biosciences), which was prerequisite for all other experiments, an aliquot of the conidia was used, washed and stained with anti-human C3c FITC antibody (Dako). FITC-labeled swollen non- or C-opsonized conidia were used for binding and internalization assays acquired by FACS and confocal microscopic analyzes. FITC labeling was performed as described elsewhere [17]. Briefly, swollen conidia were labeled using 0.1 mg/ml FITC (Sigma) in 0.1 M Na_2_CO_3_ for 30 min at 37°C. Conidia were washed several times and re-suspended in RPMI 1640 (Gibco), supplemented with 10% FCS and 1% L-Glutamine (RPMI^++^) and used immediately for all further experiments.

### Cell assays

*DCs were incubated with swollen opsonized and non-opsonized FITC-labeled WT and mutant conidia at a MOI = 2 either on ice (binding only, since DCs do not internalize conidia at this temperatures* [18]*) or at 37°C (binding and internalization) for 6h. Binding and internalization were analyzed by flow cytometry gating for FITC-positive conidia in DCs. For blocking experiments, DCs were pre-incubated with blocking Abs (DC-SIGN/DC-SIGNR, Novus Biologicals, 10µg/ml, cat 120612) prior addition of differentially opsonized conidia and analyzed by RT-PCR*. In detail, for blocking experiments, DCs were incubated with 10µg/ml α-DC-SIGN + DC-SIGNR antibody (Novus Biologicals) for 30 minutes at 37°C. Opsonized and non-opsonized swollen conidia of the WT and *fks1_tetOn_* mutant were co-cultured with DCs at MOI of 5 and cells were lysed after 3h for RNA isolation and RT-PCR for IL23A (BioRad Laboratories) was performed, as described in detail above.

Percentages of FITC-labelled conidia bound and/or internalized to DCs from at least 5 independent experiments were assessed by multi-color flow cytometry. Differences were analyzed by one Way ANOVA using Bonferroni´s posttest for multiple comparisons.

### Immunoblot analyses of phosphorylated proteins

DCs were starved in RPMI1640 containing 0.5% FCS and 1% L-Glutamine for 3h. The starving of cells was performed to set their phosphorylation to background levels. Following starvation, DCs were incubated with the differentially opsonized WT and mutant *A. fumigatus* strains at a MOI of 5. Cells were lysed after 1h co-incubation with RIPA Buffer (Sigma-Aldrich) containing protease and phosphates inhibitors and EDTA (Thermo-Scientific). The protein content was determined by BCA (Thermo-Scientific). Lysates were separated using 14% SDS-PAGE gels, transferred to PVDF membranes and incubated with anti-human a-tubulin or ERK1/2 as loading controls as well as anti-human phospho-p38, P-JNK1/2, NFκB and phospho-ERK1/2 (all from Cell Signaling Technology) and developed with the Lass 4000 Image Quant.

### Multiplex PCR analyses

DCs were infected with the differentially opsonized WT and mutant *A. fumigatus* for 3h at 37°C with a MOI of 5. Cells were lysed with the RLT Buffer (Qiagen) and total RNA was purified according to the manufacturer’s advice. RNA was then measured (NanoVue) and reverse transcribed into cDNA (iSkript Reverse Transcription Supermix for RT-qPCR, BioRad). The cDNA was then used for multiplex qPCR (iQ Multiplex Powermix, BioRad) amplification, using PrimePCR™ Probes for IL-10, IL-6, IL-1B, IL12A, IL23A and TGFB (all from BioRad Laboratories). The RT-qPCR was run in the BioRad CFX96 Real Time PCR System. Data were analyzed with the BioRad CFX Manager Software and values were exported to GraphPad Prism.

### Confocal microscopy

To study the interaction of swollen, opsonized and non-opsonized conidia with CRs on DCs, cells were incubated with conidia at a 1:2 ratio. Confocal slides were coated with Poly-L-Lysine (Sigma-Aldrich) for 2h at 37°C. DCs were stained with Hoechst, anti-human CD11b- or CD11c-Alexa594 and anti-human DC-SIGN Alexa633 (all from BD) and added to the PBS washed slides for at least 1h until they were adherent. FITC-labeled opsonized and non-opsonized conidia of the different strains were added to the cells for 6h to have comparable conditions as during binding/internalization experiments evaluated by flow cytometry. Pre-warmed D-PBS was used to remove unbound conidia and slides were fixed using 1% formaldehyde for 20min. Slides were mounted onto coverslips with Mowiol (Sigma) and used for microscopy. Images were acquired on a Leica SP5 (x63 Glycerin) confocal microscope at the Biooptics core facility, CCB, Innsbruck, Austria. Deconvolution was performed using Huygens Professional and images were merged using ImageJ Software.

### Incubation of DCs with soluble chitin, galactomannan and fungal supernatants

Soluble polysaccharides as chitin (Sigma-Aldrich) and galactomannan (Carl-Roth) were diluted as previously described [45,46]. DCs were incubated with chitin (10µg/ml), galactomannan (20µg/ml) or fungal supernatants. WT and *fks1_tetOn_^rep^* were grown in a concentration of 25 × 10^8^ spores in 200ml AMM for 24h. The supernatants were centrifuged and sterile-filtered prior to addition at a ratio 1:10 to the cells, which represent a MOI of 5. Cells were lysed after 1h for Immunoblot analysis and after 3h co-culture for multiplex PCR analysis, as described above in detail.

### Statistical analyses

Differences were analyzed by the GraphPad prism software using the unpaired student’s t-test (2-tailed) or one-way ANOVA with Bonferroni posttest for multiple comparisons depending on the analyzes.

## Supplementary Material

Supplemental Material
