# Supplementary material for: β-1,3-glucan-lacking Aspergillus fumigatus mediates an efficient antifungal immune response by activating complement and dendritic cells
Source: Virulence. 2018 Oct 29;10(1):957–69. doi: 10.1080/21505594.2018.1528843 (PMC8647855; doi:10.1080/21505594.2018.1528843)

Supplementary Data

Suppl. Fig. 1 Involvement of CR4 in internalization of C-opsonized conidia. Not only CR3, but also CR4 participates in the up-take of opsonized fungal conidia (green) devoid of either DHN-melanin (*ΔpksP-C)* or β-1,3-glucan (*fks*$_{tetOn}^{rep}$-C) as detected by fluorescent staining of the CR4 α-chain, CD11c (yellow). DC-SIGN (red) co-localized with germinating fungi (arrows in the *fks*$_{tetOn}^{rep}$ panel). Nuclei are stained in blue using Höchst. As already observed for CR3 staining experiments, complement-opsonization of fungal conidia caused an accumulation of the spores within DCs independent on the cell surface (2nd and 4th panel) compared to their non-opsonized counterparts (1st and 3rd panel). An overview of about 10 cells per condition is illustrated in this figure. Scale bar represents 10µm.

Suppl. Fig.1

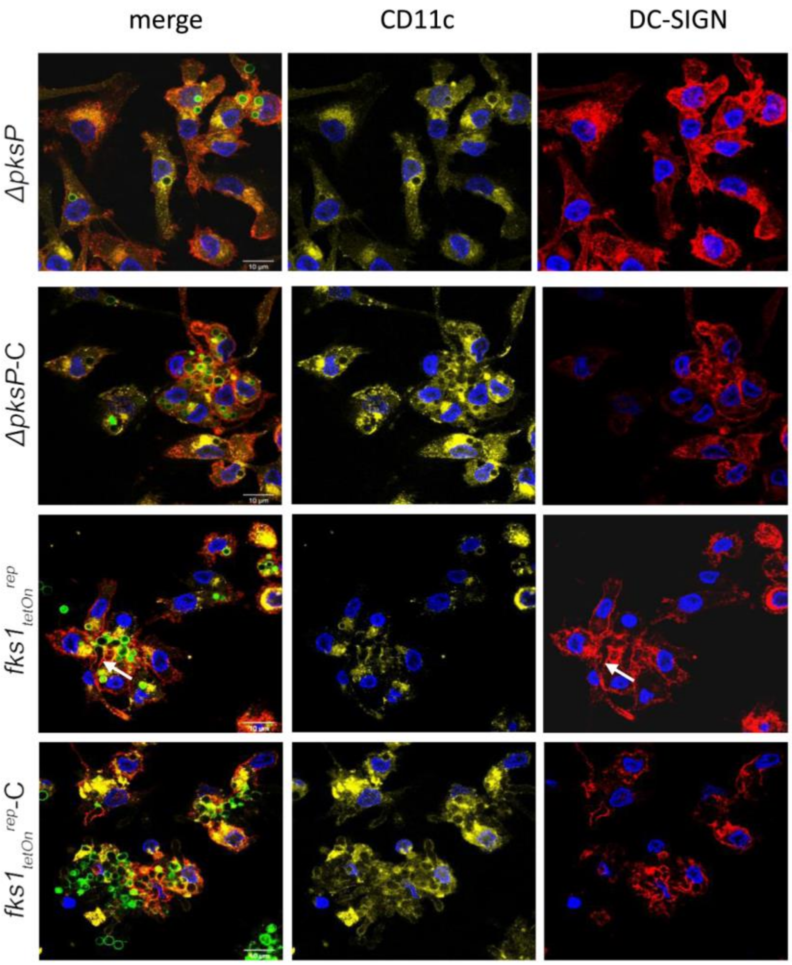

Supplementary Figure 2 A pro-inflammatory cytokine pattern is induced in DCs exposed to $fks1_{tetOn}^{rep}$ SN. Real-time PCR analyses revealed that IL1B (upper, left), IL6 (lower, left), and IL23A (upper, right) mRNA expression are elevated to significantly higher levels in DCs exposed to the SN of $fks1_{tetOn}^{rep}$ compared to its respective WT AfS35. IL10 mRNA expression levels were not or only slightly changed by treatment with SNs from AfS35, $fks1_{tetOn}^{rep}$ or galactomannan (20 µg/ml) compared to chitin (10 µg/ml)-treated DC controls. One representative donor out of three is shown and statistical analyses were performed using GraphPad Prism Software.

Suppl. Fig.2

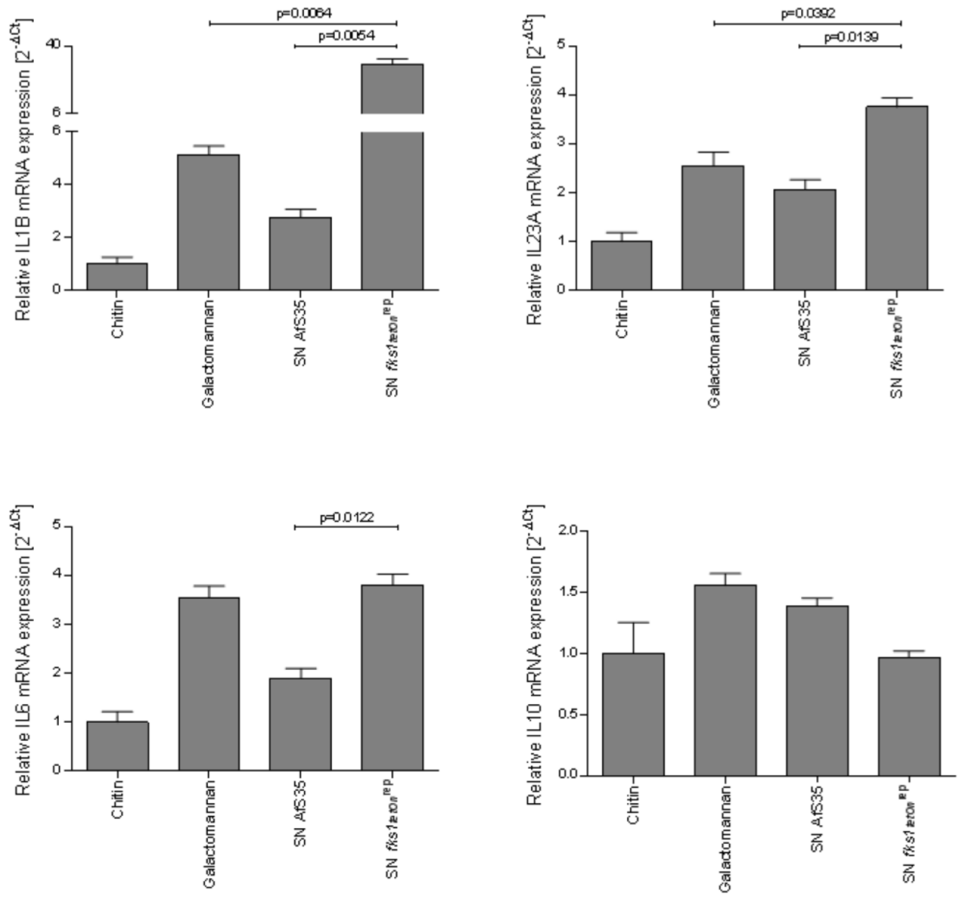

Supplement: Supplemental Material [file KVIR_A_1528843_SM6458.zip › Supplementary Data.pdf]
