# Supplementary figures and images for: β-1,3-glucan-lacking Aspergillus fumigatus mediates an efficient antifungal immune response by activating complement and dendritic cells
Source: Virulence. 2018 Oct 29;10(1):957–69. doi: 10.1080/21505594.2018.1528843 (PMC8647855; doi:10.1080/21505594.2018.1528843)

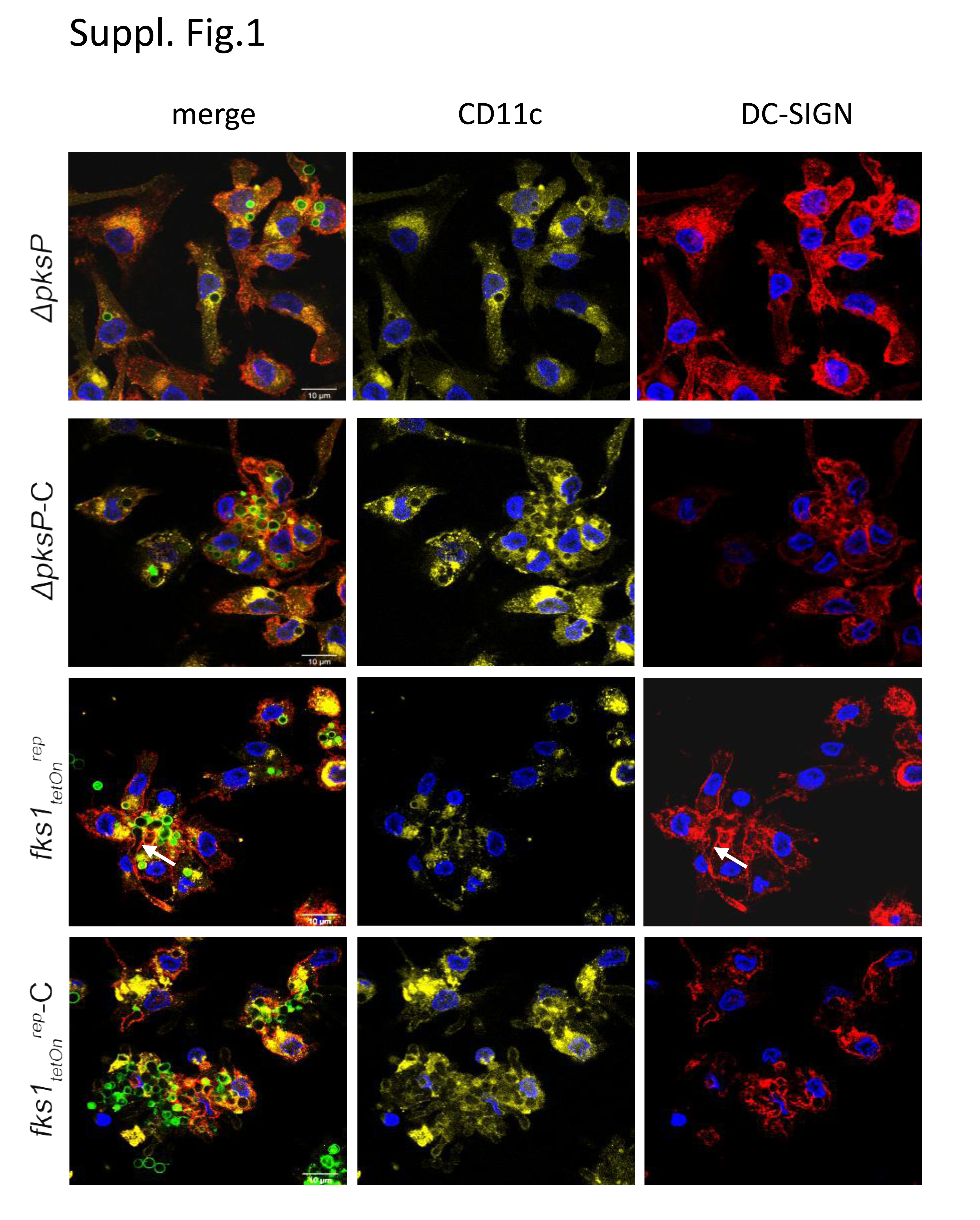

Supplement: Supplemental Material [file KVIR_A_1528843_SM6458.zip › Suppl Figure 1.tif]

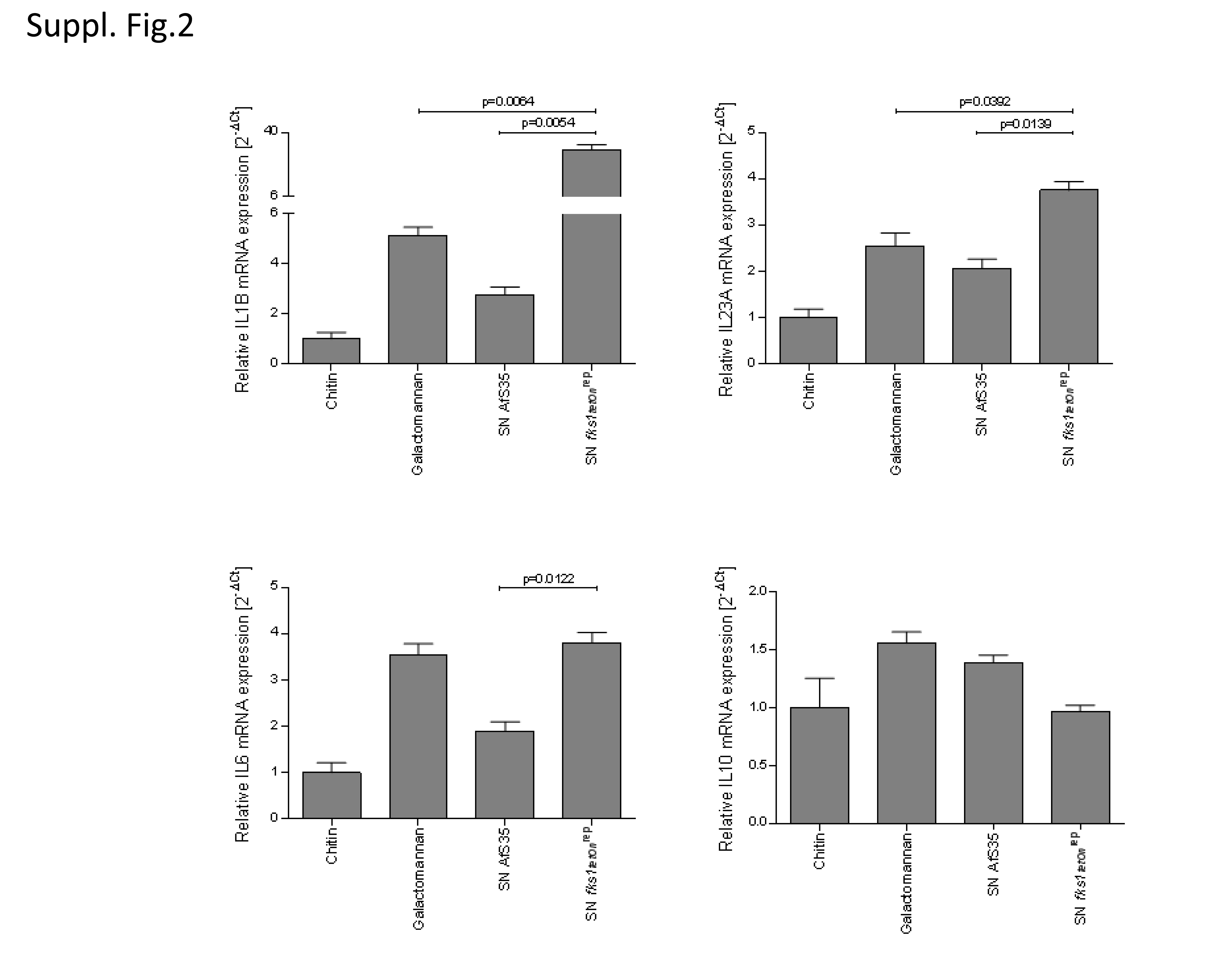

Supplement: Supplemental Material [file KVIR_A_1528843_SM6458.zip › Suppl Figure 2.tif]

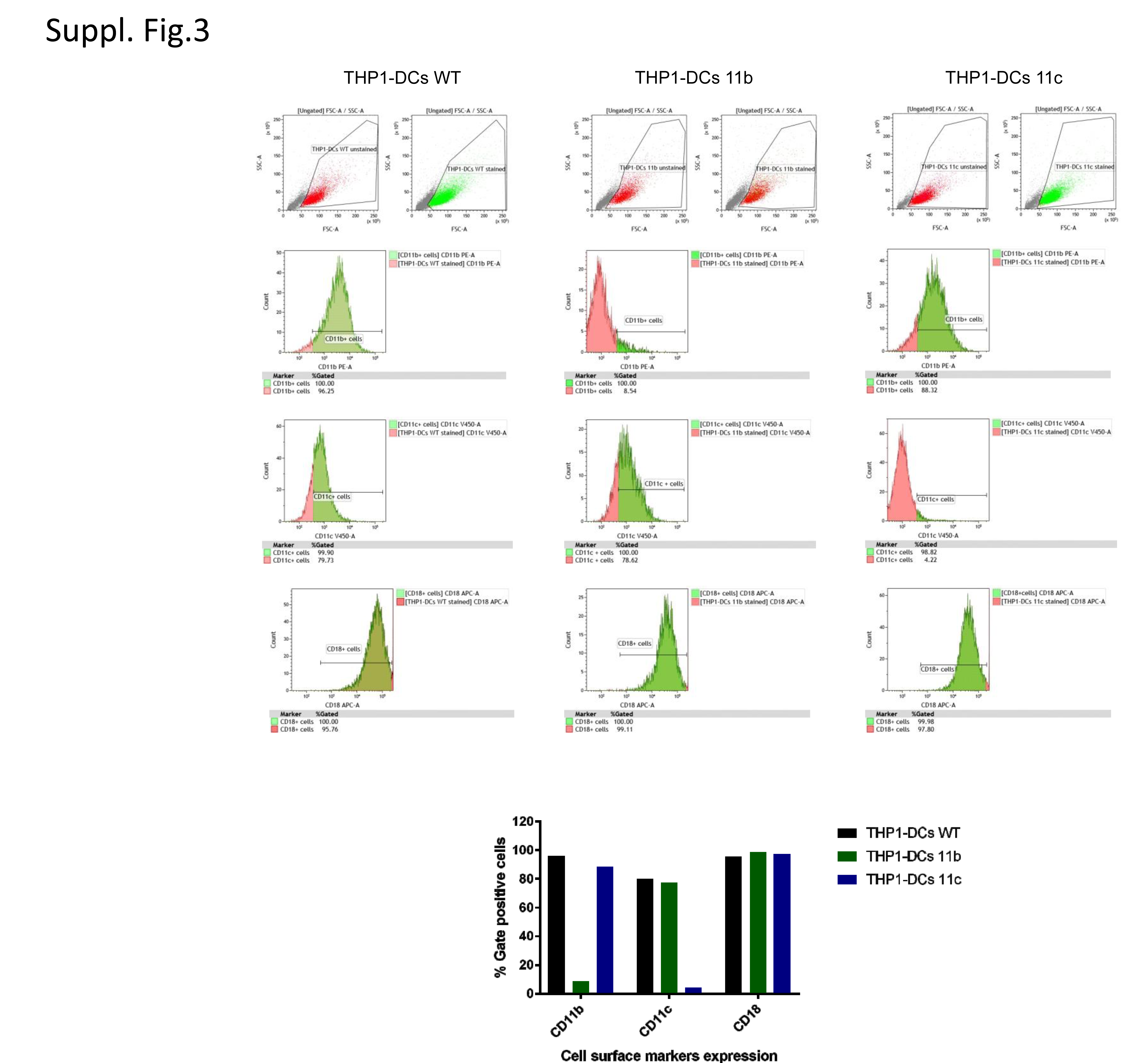

Supplement: Supplemental Material [file KVIR_A_1528843_SM6458.zip › Suppl Figure 3.tif]
